# Genomic Analysis of *Enterobacter* Species Isolated from Patients in United States Hospitals

**DOI:** 10.3390/antibiotics13090865

**Published:** 2024-09-10

**Authors:** Fred C. Tenover, Isabella A. Tickler

**Affiliations:** 1 College of Arts and Sciences, University of Dayton, Dayton, OH 45469, USA; ftenover1@udayton.edu; 2 Cepheid, Sunnyvale, CA 94089, USA

**Keywords:** *Enterobacter*, sequencing, AmpC beta-lactamases, carbapenemases, antimicrobial resistance

## Abstract

We analyzed the whole genome sequences (WGS) and antibiograms of 35 *Enterobacter* isolates, including *E. hormaechei* and *E. asburiae*, and the recently described *E. bugandensis*, *E. kobei*, *E. ludwigii*, and *E. roggenkampii* species. Isolates were obtained from human blood and urinary tract infections in patients in the United States. Our goal was to understand the genetic diversity of antimicrobial resistance genes and virulence factors among the various species. Thirty-four of 35 isolates contained an AmpC class *bla_ACT_* allele; however, the *E. roggenkampii* isolate contained *bla_MIR-5_*. Of the six *Enterobacter* isolates resistant to ertapenem, imipenem, and meropenem, four harbored a carbapenemase gene, including *bla_KPC_* or *bla_NDM_*. All four isolates were mCIM-positive. The remaining two isolates had alterations in *ompC* genes that may have contributed to the resistance phenotype. Interpretations of cefepime test results were variable when disk diffusion and automated broth microdilution results were compared due to the Clinical Laboratory and Standards Institute use of the “susceptible dose-dependent” classification. The diversity of the *bla_ACT_* alleles paralleled species identifications, as did the presence of various virulence genes. The classification of recently described *Enterobacter* species is consistent with their resistance gene and virulence gene profiles.

## 1. Introduction

The genus *Enterobacter* consists of human, animal, and plant pathogens as well as environmental species [1]. *Enterobacter* species are included among the “ESKAPE” pathogens, i.e., as key causes of healthcare-associated infections that are often multidrug-resistant [1,2,3]. The taxonomy of the genus *Enterobacter* is complex and fluid, consisting of multiple individual species, subspecies, and species complexes. Several longstanding human pathogens, e.g., the former species *Enterobacter aerogenes* and *Enterobacter sakazakii*, have been reclassified as *Klebsiella aerogenes* and *Cronobacter sakazakii*, respectively [4,5,6]. *Enterobacter* species typically produce potent AmpC beta-lactamases capable of hydrolyzing most extended-spectrum cephalosporins, with the exception of the fourth-generation cephalosporin, cefepime. AmpC beta-lactamases, together with changes in the outer membrane porins OmpF and OmpC, have been reported to mediate carbapenem resistance in *Klebsiella aerogenes*, and likely in *Enterobacter* species as well [1,7]. Several *Enterobacter* species have been reported to produce carbapenemases, including NDM, KPC, and OXA-48, in addition to less common carbapenemases, such as NMC/IMI [3,8]. The AmpC enzymes of *Enterobacter* species may give false positive results for some phenotypic susceptibility testing methods, such as Modified Hodge tests to detect carbapenemase production [9]. The antimicrobial resistance profiles of *Enterobacter* species often include resistance to multiple classes of antimicrobial agents, including aminoglycosides and fluoroquinolones, which can make the treatment of human infections with these organisms difficult [10].

Our preliminary analysis of a recent collection of *Enterobacter* isolates from the United States revealed discrepancies between the species identifications produced by whole genome sequencing (WGS) methods and by MALDI-TOF. However, newer databases for both WGS data and MALTI-TOF have become available. Thus, we wanted to determine whether the newer databases would resolve the discrepancies in species identification. Furthermore, we sought to determine whether the newer species identifications would contain novel AmpC enzymes and virulence factors. Consequently, the goals of this study were to analyze the WGS data and antibiograms of the *Enterobacter* isolates obtained from human infections in patients in the United States to determine (1) how sequence-based species identification results differed from identifications by MALDI-TOF, (2) how AmpC-type beta-lactamases varied by species, (3) whether differences among the AmpC enzymes were reflected in varying susceptibilities to beta-lactam antibiotics, and (4) whether virulence factors were consistent among the different *Enterobacter* species.

## 2. Materials and Methods

Bacterial strains: A convenience sample of 35 isolates of a variety of *Enterobacter* species from patients in U.S. hospitals in 11 different states was selected from our reference collection for the study. Selection criteria included isolates that were non-susceptible to cefotaxime, ceftriaxone, ceftazidime, ertapenem, meropenem, or imipenem, as previously described [11].

Bacterial identification: Organisms were identified originally using MALDI-TOF MS (Bruker Daltonics GmbH, Bremen, Germany) RUO Library revisions G (2020) and H (2021), and by WGS data analysis using K-mer spectra analysis (the kmer database for species identification was downloaded on 24 May 2023) and the NCBI average nucleotide identity (ANI) workflows [11,12]. Identifications were subsequently repeated using updated versions of the two databases, specifically the MALDI-TOF MBT Compass (RUO) Library revision K (2022) and the new K-mer spectra analysis database (downloaded on 24 May 2024).

Antimicrobial Susceptibility Testing: Antimicrobial susceptibility testing (AST) was performed using an automated broth microdilution system (ABMD) with the MicroScan Walkaway Neg MIC 56 panels (Beckman Coulter Inc., West Sacramento, CA, USA) and by disk diffusion (DD) following Clinical Laboratory and Standards Institute (CLSI) guidance and interpreted using CLSI criteria. Both CLSI and European Committee on Antimicrobial Susceptibility Testing (EUCAST) interpretative criteria were used for cefepime results when discrepancies in interpretations specifically for this antimicrobial agent became apparent [13,14,15]. Carbapenemase activity was detected using the mCIM and eCIM tests as described in the CLSI M100 document [14].

Whole genome sequencing: Nucleic acid extraction, sequence assembly, and whole genome sequencing analysis were performed as previously described using the CLC Genomics Workbench version 22.0.2 and CLC Microbial Genomics Module version 22.1.1 (QIAGEN Bioinformatics, Aarhus, Denmark) [11]. Multi-locus sequence typing (MLST, carried out using the 7-locus scheme), k-mer based prediction of species, and the detection of acquired antimicrobial resistance genes were performed using workflows from the CLC Microbial Genomics Module version 22.1.1 (QIAGEN Bioinformatics). The ResFinder database (downloaded on 1 March 2024) was used for the detection of acquired resistance genes in the CLC Microbial Genomics Module workflow. A phylogenetic tree of the *Enterobacter* spp. strains in this study was inferred using CSIPhylogeny, the single nucleotide polymorphisms (SNP) analysis tool created by the Center for Genomic Epidemiology (https://www.genomicepidemiology.org/services/ accessed on 24 April 2024) [16]. Nucleic acid sequence data from this study have been deposited in GenBank under BioProject Accession # PRJNA981469. 

Analysis of *Enterobacter hormaechei* from Pathogen Detection collection: *E. hormaechei* sequences were downloaded from the NCBI Pathogen Detection collection (https://www.ncbi.nlm.nih.gov/pathogens/isolates accessed on 30 August 2024) to compare sequence types and beta-lactamase genes with the dataset from the current study. For a closer comparison, the query was restricted to a creation date of 2021-2022, United States-only, and isolation source blood and urine. A total of 48 assemblies were downloaded. Multi-locus sequence typing (MLST, carried out using the 7-locus scheme) and the detection of acquired antimicrobial resistance genes were performed using workflows from the CLC Microbial Genomics Module version 22.1.1 (QIAGEN Bioinformatics). The ResFinder database (downloaded on 1 March 2024) was used for the detection of acquired resistance genes in the CLC Microbial Genomics Module workflow.

## 3. Results

### 3.1. Enterobacter Species Identification and MLST Typing

The initial identification of the isolates by MALDI-TOF yielded one isolate of *E. asburiae*, one *Enterobacter bugandensis*, two *Enterobacter hormaechei*, three *Enterobacter xiangfangensis*, and twenty-eight belonging to the *Enterobacter cloacae* complex. A comparison of the results of the three identification methods, two based on WGS (ANI and kmer) and the updated MALDI-TOF database, showed significant changes in the identifications and indicates agreement to the species level among all methods used for the 35 isolates (Table 1). However, the k-mer spectra WGS method added subspecies identifications to 28 of the *E. hormaechei* isolates.

**Figure 1:**
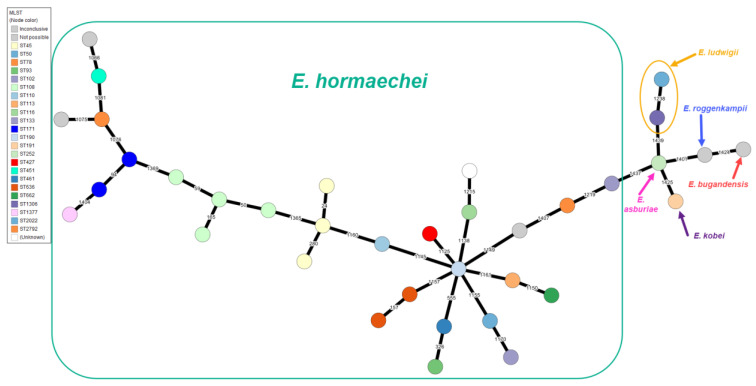
Minimum Spanning Tree based on MLST of *Enterobacter* spp. Node color indicates the sequence type (ST) identified. Squares, circles, and arrows indicate species identified by ANI. Reference strain (REF) is *Enterobacter hormaechei* subsp. *steigerwaltii* strain VKH10 (Accession ASM2421883v1).

Overall, 23 distinct MLST types were identified among all isolates. Among the 29 isolates now identified as *E. hormachei*, there were 19 different MLST types, with an additional 3 isolates showing inconclusive MLST results (Figure 1). Among the *E. hormaechei* isolates, small clusters of related MLST types could be identified, such as two isolates belonging to the successful ST171 lineage, typically associated with *E. hormaechei* subsp. *xianfangensis*, one from New Jersey and the other from Kansas (Figure 1 and Table S1). A cluster of four strains belonging to ST108 were obtained from three geographically distinct hospitals, but all carried *bla_ACT-55_*. Lastly, two groups of genetically related isolates (three ST45 and two ST636) were again from geographically distinct hospitals, suggesting that these clones are widely dispersed in the U.S. (Figure 1 and Table S1).

**Figure 2:**
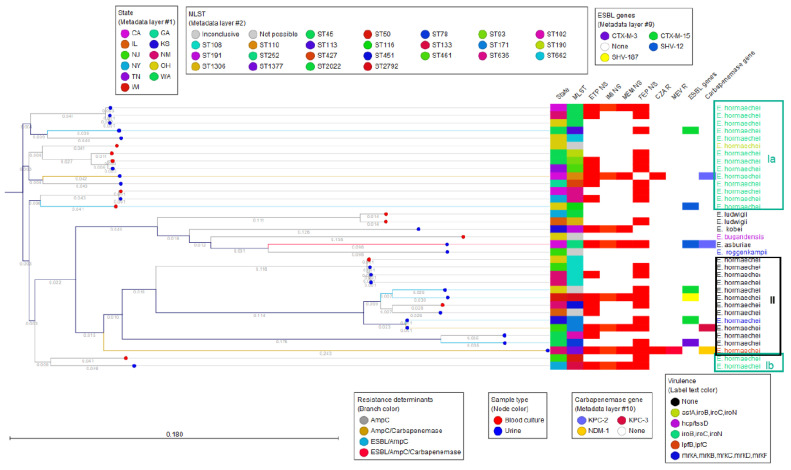
SNP tree constructed using the CGE CSI Phylogeny tool. Reference strain used was *Enterobacter hormaechei* spp. *steigerwaltii* ASM2421883v1. The three clusters of *E. hormaechei* designated as Ia and Ib (green) and II (black) are shown.

The *Enterobacter* spp. phylogenetic tree inferred with the CGE SNP tree method showed the 29 *E. hormaechei* isolates separated into two major clusters (named I and II) with distinct characteristics in their virulence genes (Figure 2). Cluster I was further subdivided into clusters Ia and Ib based on sequence divergence (Figure 2). This divergence and clustering within *E. hormaechei* are also evident in the Minimum Spanning Tree constructed with MLST data (Figure 1).

### 3.2. Phenotypic Antimicrobial Susceptibility Testing Results for Beta-Lactams

All of the isolates were resistant with ABMD to ampicillin, cefazolin, cefoxitin, and amoxicillin–clavulanic acid, with the exception of one isolate that was intermediate to the latter drug (MIC = 16/8 µg/mL). Thus, there were no unique phenotypes apparent among the *Enterobacter* species with regard to these antimicrobial agents. Overall, the interpretations of the ABMD results were consistent with DD results, except for cefepime. Cefepime showed the greatest variation in interpretations of AST results among the antimicrobial agents tested, in part due to the susceptible dose-dependent (SDD) designation used exclusively by CLSI. Thus, for cefepime, we also compared the categorical results using both CLSI and EUCAST criteria (Table S1). Twelve isolates were susceptible to cefepime according to all four criteria (ABMD and DD results using both CLSI and EUCAST criteria). Thirteen isolates were resistant to cefepime by ABMD testing using both CLSI and EUCAST criteria; all thirteen but one (that was intermediate) were also resistant using EUCAST DD criteria (CLSI and EUCAST used the same concentration of cefepime in the disk). However, of those cefepime-resistant or intermediate isolates by DD, six were called SDD by CLSI. The remaining 10 isolates (out of 35 *Enterobacter* spp.) tested by ABMD were called SDD by CLSI criteria but interpreted as resistant or intermediate by EUCAST criteria. Four of the ten were interpreted as susceptible by CLSI DD and just one was susceptible according to EUCAST DD, while the rest were either resistant or susceptible. The ABMD and DD results were interpreted as SDD by CLSI criteria, but R by EUCAST breakpoints included an *E. hormaechei* harboring *bla_KPC-2_*. Overall, EUCAST criteria tended to show more resistant results than CLSI.

### 3.3. Phenotypic and Genotypic Results for Aminoglycosides and Fluoroquinolones

Nine isolates (eight *E. hormaechei* and one *E. asburiae*) were tobramycin-resistant, which was consistent with the presence of *aac(6′)-Ib-cr* gene. Only three isolates were gentamicin-non-susceptible (two resistant and one intermediate), which correlated with the presence of the *ant(2′)-Ia*, *aac(3)-IIa*, and *aac(3)-IVa* genes.

Ciprofloxacin resistance was seen in ten isolates, including six with *aac(6′)-Ib-cr* with or without a *qnr* gene, two with only *qnrS1*, and two with no acquired fluoroquinolone resistance but with well-described alterations in the chromosomal *gyrA* at positions S83F and D87A, and in *parC* at position S80I. All the mutations have previously been associated with fluoroquinolone resistance [17]. Eight of the ten isolates were also levofloxacin-resistant, while one was intermediate and one, which was carrying the *qnrS1* gene, was susceptible (Table S1). Both aminoglycoside and fluoroquinolone resistance appeared more frequently in the *E. hormaechei* isolates of cluster II of the SNP tree (Figure 2). There was no correlation with specific beta-lactamase resistance gene carriage and the cefepime resistance phenotype.

### 3.4. AmpC Diversity

**Figure 3:**
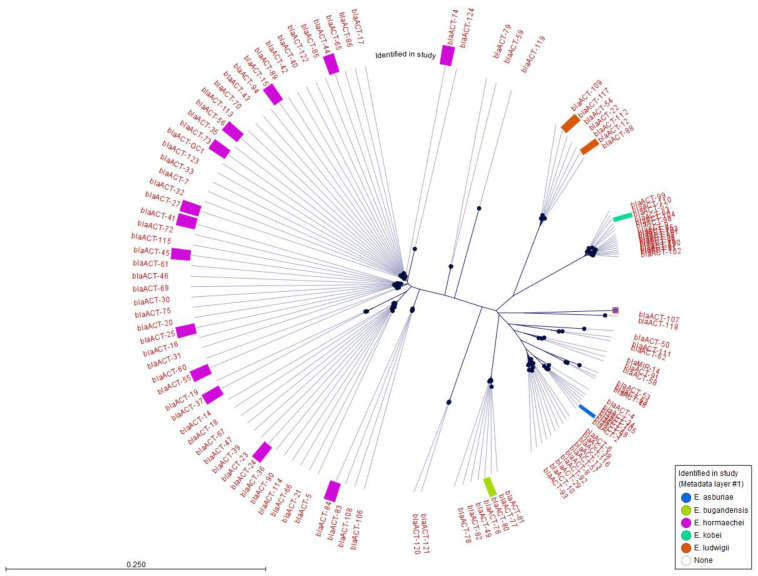
Maximum Likelihood Phylogeny of all bla_ACT_ genes. The *bla_ACT_* genes identified in the study and the species carrying them are distinguished by colored bands.

AmpC beta-lactamase genes were identified in all of the *Enterobacter* isolates. Thirty-four of thirty-five isolates contained a *bla_ACT_* allele; however, the *E. roggenkampii* isolate (17,310) contained a different AmpC-type beta-lactamase, *bla_MIR-5_* (with 99.48% sequence identity to the *bla_MIR-5_* reference gene). A BLAST search of the contig containing the genomic environment surrounding the *bla_MIR-5_* gene of isolate 17,310 also returned a 99% match to the chromosome of a published *E. roggenkampii* strain (GenBank accession CP056737.1). Eighteen different *bla_ACT_* genes were detected among the thirty-four isolates (Figure 3). Among the 29 isolates identified as *E. hormaechei* by ANI, 14 different *bla_ACT_* genes were detected, although many clustered in highly related branches (Figure 3). The two *E. ludwigii* isolates had different but highly related *bla_ACT_* alleles (*bla_ACT-12_* and *bla_ACT-117_*; Figure 3). The *bla_ACT-80_* from *E. bugandensis* and the *bla_ACT-52_* from *E. kobei* were on distinct branches of the *bla_ACT_* phylogenetic tree, while the *bla_ACT-3_* from *E. asburiae*, while also on a distinct branch, was more closely linked to other *bla_ACT_* alleles (Figure 3). Thus, the presence of specific *bla_ACT_* alleles was aligned with their *Enterobacter* species host.

### 3.5. ESBLs and Other Beta Lactamases

Fifteen isolates contained beta-lactamase genes in addition to the *bla_ACT_* genes. This included seven isolates with narrow-spectrum beta-lactamase genes (*bla_TEM-1B_*, *bla_OXA-1_*, *bla_OXA-9_*, or *bla_OXA-10_*), two of which also carried a carbapenemase gene, seven isolates with ESBL genes (*bla_CTX-M-3_*, *bla_CTX-M-15_*, *bla_SHV-7_* and *bla_SHV-12_*), plus or minus a narrow-spectrum beta-lactamase or a carbapenemase gene, and one isolate with a *bla_KPC-2_* in addition to the *bla_ACT-27_* gene. Of the seven isolates with ESBLs and AmpC beta-lactamases, four were identified as ESBLs by cefotaxime-clavulanic acid testing, ceftazidime-clavulanic acid testing, or both. Four isolates that did not contain an ESBL but did have a *bla_ACT_* allele were falsely positive with a clavulanic acid combination test. One isolate with *bla_KPC_* was also identified as positive by clavulanic acid combination testing (Table S1).

### 3.6. Carbapenem Resistance Phenotypes and Genotypes

There were 16 isolates that were resistant to ertapenem according to ABMD (15 *E. hormaechei* and 1 *E. asburiae*), and 1 additional isolate that was intermediate (*E. kobei*). All 17 isolates were also resistant or intermediate to ertapenem according to DD testing. Six of the isolates (five *E. hormaechei* and one *E. asburiae*) were also resistant to meropenem and imipenem according to ABMD and DD, and two additional isolates were intermediate to meropenem. However, only one *E. hormaechei* was resistant to the meropenem–vaborbactam combination. Of the six *Enterobacter* isolates resistant to ertapenem, imipenem, and meropenem, four harbored a carbapenemase gene. The carbapenemase genes detected by WGS included two *bla_KPC-2_* (one in *E. hormachei* and one in *E. asburiae*), one *bla_KPC-3_* (in *E. hormaechei*), and one *bla_NDM-1_* (in *E. hormaechei*). All four isolates were mCIM-positive, and the positive eCIM result for the *bla_NDM-1_*-containing isolate was consistent with a metallo-beta-lactamase. Both *bla_KPC-2_* producers harbored a pKPC-CAV1193 plasmid and were from the same hospital, although the genes were from different *Enterobacter* species, suggesting that the plasmid was transmissible. The two carbapenem-resistant *E. hormaechei* isolates that did not carry a carbapenemase gene had either a single amino acid substitution or an amino acid deletion in *ompC*, which was not found among the other isolates. Reduced carbapenem susceptibility mediated by non-enzymatic mechanisms such as porin alterations has been described in *Enterobacter* spp. [18,19].

### 3.7. Virulence Genes

Virulence genes were identified by WGS to better understand the genetic diversity of the various *Enterobacter* species. The siderophore-encoding gene cluster *iroB/iroC/iroN* was detected in 16 out of 35 isolates (45.7%), all of which were *E. hormaechei*, and all of which belonged to both phylogenetic clusters, Ia and Ib (Figure 2). One of the isolates also carried the *astA* gene, which encodes a heat-stable enterotoxin. The *E. bugandensis* carried type VI secretion system (T6SS)-associated genes *hcp/tssD* [20,21]. Among phylogenetic cluster II, only two isolates carried known virulence genes. One *E. hormaechei* isolate, belonging to ST1377, harbored the *lpfB*, *lpfC* genes, which encode long polar fimbriae. The *mrk* gene cluster (*mrkA*, *mrkB*, *mrkC*, *mrkD*, *mrkF*), encoding type 3 fimbrial adhesins, was found in one *E. hormaechei* and one *E. roggenkampii* [22] (Figure 2).

### 3.8. Pathogen Detection of E. hormaechei

The NCBI Pathogen Detection (PD) query and typing results are listed in Supplementary Table S2. Twenty-six unique sequence types were identified out of the forty-eight sequences of E. hormaechei isolates obtained from blood and urine clinical samples. ST190 was the most frequent sequence type, representing 14.6 % (7/48) of all strains, followed by ST461 and ST78 (both 8.3%, 4/48). These sequence types were also identified in the current study. However, ST108, the most frequent MLST observed in our study, was not identified in the PD dataset. Similarly, there were 27 sequence types that did not overlap between the two collections of E. hormaechei. Overall, 15 bla_ACT_ subtypes were identified, with bla_ACT-73_ representing 35.4% (17/48) of the sequences in the dataset, followed by bla_ACT-56_ (14.6%, 7/48). In the current study, bla_ACT-73_ is also the predominant subtype, and was harbored by 20.7% of all E. hormaechei (6/29), while bla_ACT-56_ was carried by 10.3% (3/29). Carbapenemase genes were identified in 43.7% (21/48) of the PD strains, as opposed to the 10.3% (3/29) observed among E. hormaechei in the current study (Table S1 and Table S2).

## 4. Discussion

We sequenced 35 *Enterobacter* isolates from human blood and urinary tract infections to understand the genetic diversity of antimicrobial resistance genes and virulence factors, especially among the recently described *Enterobacter* species. Sutton et al. reported that many bacterial isolates identified as *E. cloacae* complex (ECC) by non-sequence-based methods are often incorrectly classified [4]. Most of these isolates are *E. hormaechei* or subspecies of *E. hormaechei* when identified by WGS methods, such as those we characterized in this study [4]. Interestingly, we also noted that many of our isolates, originally identified as ECC by MALDI-TOF, changed to *E. hormaechei* when retested with a newer MALDI-TOF database. According to the MALDI Biotyper Update Release notes (Revision K, 2022. Ref. 1829023), a new set of 48 fully characterized ECCs was added to the library, resulting in 69 ECCs now being identified at the species level as opposed to the complex level (e.g., *E. roggenkampii*). This illustrates both the confusion that microbiologists and physicians often face when dealing with infection caused by *Enterobacter* species and the importance of updating databases frequently [23,24]. Even when sequence-based methods are used, species identification can vary by method, as we have noted here. The biggest difference between the WGS-based identifications was the addition of subspecies names to all but one of the *E. hormaechei* isolates by the Kmer method, as opposed to the simpler identification of *E. hormaechei* given by ANI predictions. As noted by Sutton and colleagues, when using the nomenclature established by Hoffman et al., “…genomes named *E. xiangfangensis* in GenBank fell within the *E. hormaechei* subsp. *steigerwaltii* cluster rather than a separate cluster. Moreover, most of the genomes in these clusters were mistakenly identified as *E. cloacae* when they were submitted to GenBank.” [4]. The polyphyletic nature of *Enterobacter* described by Chavda et al. was evident in our small collection of isolates, as shown by the SNP phylogenetic tree, where *E. hormaechei* isolates clearly diverge into three branches [25]. Three genetic clusters of *E. hormaechei*, one with higher divergence and two more closely related, but all still assigned to the same species, had originally been described by Hoffmann et al. [26]. Identifications of *Enterobacter* isolates at the species or subspecies level will no doubt continue to be a challenge in the future.

The diversity of the MLST profiles, especially among *E. hormachei* isolates, was surprising. Ironically, while several well-known MLST types were present in multiple hospitals, the strains were apparently not associated with outbreaks of infections within those hospitals. Thus, the epidemiology of *Enterobacter* infections indicates a broad dispersion of strain types but few clusters of infections. This rich diversity of MLST types was observed in the similar dataset (same year, country, and sample types) downloaded from Pathogen Detection, where not only were common sequence types observed between the current study and the PD dataset, but also over 20 unique sequence types.

The diversity of the chromosomal AmpC beta-lactamases, which in 34 of 35 isolates were *bla_ACT_* enzymes, was also surprising. The sole *E. roggenkampii* isolate carried a *bla*_MIR-5_ but no *bla*_ACT_. Although *bla_MIR-1_* was initially reported to have been derived from an *E. cloacae* AmpC gene [27], a subsequent study concluded that it more likely originated from *E. roggenkampii*, which is consistent with our data [28]. The phylogenetic tree of the *bla_ACT_* alleles, annotated with the corresponding *Enterobacter* species identified in our study, showed that the *bla_ACT_* subtypes tended to be species-specific, as previously suggested by Dong et al. [29]. This was also confirmed by the comparison with the Pathogen Detection dataset, which showed similar *bla*_ACT_ diversity and dominant subtypes among *E. hormaechei*.

While 16 isolates in our study were resistant to ertapenem, only 6 isolates were additionally resistant to both imipenem and meropenem. Of those six, only four isolates (all from hospitals in different U.S. states) carried carbapenemases; there were three *bla_KPC_* and one *bla_NDM_*. The other two isolates, which only carried a *bla_ACT_* enzyme and a *bla_ACT_* and *bla_SHV-7_*, respectively, also harbored altered OmpC proteins that may explain the carbapenem resistance in the absence of carbapenemase genes. There were no unique susceptibility profiles associated with specific AmpC enzymes, diminishing the effectiveness of phenotypic AST to elucidate possible genetic mechanisms.

Analysis of the extended antibiograms of the various *Enterobacter* species showed a high correlation of ABMD and DD results, except for cefepime. The classification of SDD (susceptible dose-dependent) by CLSI for cefepime was reported in multiple isolates that were resistant according to EUCAST breakpoints and included a carbapenemase-producing isolate. This discrepancy in cefepime susceptibility reports, particularly among carbapenemase-producing Enterobacterales, has been reported extensively, culminating with a recommendation in the 34th edition of CLSI document M100 to suppress the resistant cefepime S/SDD results for carbapenemase producers or report them as resistant [14,30]. We did not observe any unique antimicrobial resistance patterns among other drug classes, such as aminoglycosides or fluoroquinolones. 

ESBL testing with cefotaxime and ceftazidime with and without clavulanic acid using the disk diffusion method was not a reliable indicator of ESBLs in all *Enterobacter* species, especially with the ceftazidime/clavulanic acid combination. This phenomenon has been previously reported for organisms harboring *bla*_KPC_ and/or plasmid-mediated AmpC beta-lactamases, as clavulanic acid may induce AmpC beta-lactamases [31].

The most common virulence genes found among the *Enterobacter* isolates belonged to the *iroBCDEN* gene group, which encodes siderophores also known as salmochelins [32]. Strains carrying this cluster also appeared to be phylogenetically more closely related. One ST1377 *E. hormaechei* isolate harbored *lpfB*, *lpfC* genes encoding long polar fimbriae. The same virulome was also reported in an *E. hormaechei* ST1377 with the same MLST in a study from Nepal [33]. The presence in two urine isolates of the *mrk* gene cluster, encoding type 3 fimbrial adhesins, which aid in colonization and biofilm formation, further confirms the flexibility and adaptability of the *Enterobacter* genus [22].

This study had several limitations, in particular the relatively small sample size. This limitation was in part mitigated by the fact that the bacterial isolates came from 11 distinct and geographically separated laboratories and by being able to compare and corroborate results with genomic sequences from NCBI Pathogen Detection. It would be interesting to repeat the study in the future to monitor phenotypic and genotypic changes in *Enterobacter* species isolated from urine and bloodstream infections.

## 5. Conclusions

In summary, our study further confirms the polyphyletic and dynamic nature of *Enterobacter* species and the challenges of identifying this organism at the species and subspecies level. A strong correlation of species identifications by WGS-based methods and MALDI-TOF was only achieved after updating all databases, although phylogenetic analysis revealed distinct clusters within *E. hormaechei*, suggesting that more species or subspecies-level taxonomic reclassifications may be coming. The diversity of the *bla_ACT_* alleles paralleled species identifications, as did the presence of various virulence genes. Thus, the classification of recently described *Enterobacter* species within the *E. cloacae* complex is consistent with their resistance gene and virulence gene profiles.

## Figures and Tables

**Table 1:** Identification of the 35 *Enterobacter* spp. isolates by WGS and MALDI-TOF methods.

Isolate	Isolated from	Organism by K-mer Spectra	Identification by ANI (GenBank)	Identification by MALDI-TOF *
17193	Blood culture	*Enterobacter hormaechei* subsp. *steigerwaltii*	*Enterobacter hormaechei*	*Enterobacter hormaechei* *
17317	Blood culture	*Enterobacter hormaechei* subsp. *steigerwaltii*	*Enterobacter hormaechei*	*Enterobacter hormaechei* *
17461	Blood culture	*Enterobacter ludwigii*	*Enterobacter ludwigii*	*Enterobacter ludwigii* *
17536	Blood culture	*Enterobacter hormaechei* subsp. *steigerwaltii*	*Enterobacter hormaechei*	*Enterobacter hormaechei* **
17571	Blood culture	*Enterobacter hormaechei* subsp. *steigerwaltii*	*Enterobacter hormaechei*	*Enterobacter hormaechei* **
17597	Blood culture	*Enterobacter hormaechei* subsp. *steigerwaltii*	*Enterobacter hormaechei*	*Enterobacter hormaechei* *
17616	Blood culture	*Enterobacter hormaechei* subsp. *steigerwaltii*	*Enterobacter hormaechei*	*Enterobacter hormaechei* **
17618	Blood culture	*Enterobacter hormaechei* subsp. *steigerwaltii*	*Enterobacter hormaechei*	*Enterobacter hormaechei* *
17623	Blood culture	*Enterobacter hormaechei* subsp. *steigerwaltii*	*Enterobacter hormaechei*	*Enterobacter hormaechei* *
17627	Blood culture	*Enterobacter bugandensis*	*Enterobacter bugandensis*	*Enterobacter bugandensis*
17825	Blood culture	*Enterobacter ludwigii*	*Enterobacter ludwigii*	*Enterobacter ludwigii* *
17180	Urine	*Enterobacter hormaechei*	*Enterobacter hormaechei*	*Enterobacter hormaechei* *
17181	Urine	*Enterobacter asburiae*	*Enterobacter asburiae*	*Enterobacter asburiae* *
17208	Urine	*Enterobacter hormaechei* subsp. *steigerwaltii*	*Enterobacter hormaechei*	*Enterobacter hormaechei* *
17246	Urine	*Enterobacter kobei*	*Enterobacter kobei*	*Enterobacter kobei* *
17307	Urine	*Enterobacter hormaechei* subsp. *steigerwaltii*	*Enterobacter hormaechei*	*Enterobacter hormaechei* *
17308	Urine	*Enterobacter hormaechei* subsp. *steigerwaltii*	*Enterobacter hormaechei*	*Enterobacter hormaechei* *
17310	Urine	*Enterobacter roggenkampii*	*Enterobacter roggenkampii*	*Enterobacter roggenkampii*
17328	Urine	*Enterobacter hormaechei* subsp. *steigerwaltii*	*Enterobacter hormaechei*	*Enterobacter hormaechei* *
17437	Urine	*Enterobacter hormaechei* subsp. *steigerwaltii*	*Enterobacter hormaechei*	*Enterobacter hormaechei* *
17440	Urine	*Enterobacter hormaechei* subsp. *steigerwaltii*	*Enterobacter hormaechei*	*Enterobacter hormaechei* *
17525	Urine	*Enterobacter hormaechei* subsp. *steigerwaltii*	*Enterobacter hormaechei*	*Enterobacter hormaechei* *
17526	Urine	*Enterobacter hormaechei* subsp. *steigerwaltii*	*Enterobacter hormaechei*	*Enterobacter hormaechei* *
17529	Urine	*Enterobacter hormaechei* subsp. *steigerwaltii*	*Enterobacter hormaechei*	*Enterobacter hormaechei* *
17534	Urine	*Enterobacter hormaechei* subsp. *steigerwaltii*	*Enterobacter hormaechei*	*Enterobacter hormaechei* *
17549	Urine	*Enterobacter hormaechei* subsp. *steigerwaltii*	*Enterobacter hormaechei*	*Enterobacter hormaechei* *
17550	Urine	*Enterobacter hormaechei* subsp. *steigerwaltii*	*Enterobacter hormaechei*	*Enterobacter hormaechei* *
17559	Urine	*Enterobacter hormaechei* subsp. *steigerwaltii*	*Enterobacter hormaechei*	*Enterobacter hormaechei* *
17600	Urine	*Enterobacter hormaechei* subsp. *steigerwaltii*	*Enterobacter hormaechei*	*Enterobacter hormaechei* *
17603	Urine	*Enterobacter hormaechei* subsp. *steigerwaltii*	*Enterobacter hormaechei*	*Enterobacter hormaechei* *
17609	Urine	*Enterobacter hormaechei* subsp. *steigerwaltii*	*Enterobacter hormaechei*	*Enterobacter hormaechei* *
17714	Urine	*Enterobacter hormaechei* subsp. *steigerwaltii*	*Enterobacter hormaechei*	*Enterobacter hormaechei* *
17738	Urine	*Enterobacter hormaechei* subsp. *steigerwaltii*	*Enterobacter hormaechei*	*Enterobacter hormaechei* *
17816	Urine	*Enterobacter hormaechei* subsp. *steigerwaltii*	*Enterobacter hormaechei*	*Enterobacter hormaechei*
17844	Urine	*Enterobacter hormaechei* subsp. *steigerwaltii*	*Enterobacter hormaechei*	*Enterobacter hormaechei*

* Indicates an organism identified as *E. cloacae* complex by the previous version of the MTB library. ** Indicates *E. hormaechei* identified as *E. xianfangensis* by the previous version of the MTB library.

## Data Availability

The data presented in this study are available in this article and in the NCBI BioProject database (https://www.ncbi.nlm.nih.gov/bioproject/ Submission date 8 June 2023) with accession # PRJNA981469.

## Supplementary Materials

The following supporting information can be downloaded at: https://www.mdpi.com/article/10.3390/antibiotics13090865/s1, Supplementary Table S1: SNP tree cluster, sample type, MLST, and beta-lactamase genes detected. Supplementary Table S2: NCBI Pathogen Detection *E. hormaechei* sequences, MLST, and beta-lactamase genes detected.
